# Nano-Biochar Suspension Mediated Alterations in Growth, Physio-Biochemical Activities and Nutrient Content in Wheat (*Triticum aestivum* L.) at the Vegetative Stage

**DOI:** 10.3390/plants13172347

**Published:** 2024-08-23

**Authors:** Muhammad Yousaf Shani, Samia Ahmad, Muhammad Yasin Ashraf, Maria Nawaz, Iqra Arshad, Arslan Anjum, Francesco De Mastro, Claudio Cocozza, Zafran Khan, Nimra Gul, Gennaro Brunetti

**Affiliations:** 1 Nuclear Institute for Agriculture and Biology College (NIAB-C), Pakistan Institute of Engineering and Applied Sciences (PIEAS), Islamabad 45650, Pakistan; mmyousafshani@gmail.com (M.Y.S.); iqraarshad@gmail.com (I.A.); 2 Plant Breeding and Genetics Division, Nuclear Institute for Agriculture and Biology (NIAB), Faisalabad 38000, Pakistan; 3 Institute of Molecular Biology and Biotechnology, The University of Lahore, Lahore 54590, Pakistan; saimariaz736@gmail.com; 4 Department of Botany, University of Gujrat, Gujrat 50700, Pakistan; maria.nawaz@uog.pk; 5 Center of Agricultural Biochemistry and Biotechnology (CABB), University of Agriculture Faisalabad, Faisalabad 38000, Pakistan; arslan32572@gmail.com; 6 Department of Soil, Plant, and Food Sciences, University of Bari “Aldo Moro”, 70126 Bari, Italy; claudio.cocozza@uniba.it (C.C.); gennaro.brunetti@uniba.it (G.B.); 7 Department of Plant Breeding and Genetics, University of Agriculture Faisalabad, Faisalabad 38000, Pakistan; zafrankhanmandokhail@gmail.com (Z.K.); nimratahir122@gmail.com (N.G.)

**Keywords:** foliar treatment, nano-biochar suspension, principal component analysis, Heatmap

## Abstract

Nano-biochar is a source of blackish carbonaceous material, a prerequisite for sustainable crop productivity. By using a variety of feedstock materials, nanobiochar synthesis can be employed via pyrolysis. Therefore, a project was initiated to explore the morpho-physio-biochemical alteration at the vegetative stage of wheat crops after the foliar application of nanobiochar suspension (NBS). This investigation was conducted at the Botanical Research Area of the University of Lahore in a randomized complete block design (RCBD) arrangement, with four treatments (0, 1, 3, and 5% NBS) by maintaining three replications for each treatment using the wheat variety “Zincol”. Nano biochar suspension in above mentioned concentrations were foliarly applied at the end of tillering/beginning of leaf sheath elongation of wheat seedlings to assess the morphological changes (root length, shoot length, number of leaves, fresh biomass/plant, dry biomass/plant), physio-biochemical alterations (total free amino acids, total sugars, chlorophyll content, protein, phenols, flavonoids), and nutrient uptake (Na, K, Ca, Mg, N, P contents. Our findings indicate that the foliar application of 3% NBS yielded the most favorable results across all measured attributes. Furthermore, Treatment-4 (5% NBS) specifically improved certain traits, including leaf area, total soluble proteins, and leaf calcium content. Finally, all NBS resulted in a decrease in carotenoid and sodium content in wheat seedlings.

## 1. Introduction

Agriculture plays a crucial role in improving the economies of developing countries, such as Pakistan. It imparts 25% of gross domestic productivity and engages 65% of people as their primary income source globally in developing countries [1]. Wheat (*Triticum aestivum* L.) belongs to the family Poaceae, well known as a staple food crop cultivated in various regions globally and accounts for 30% of the total cereal yield worldwide [2]. It is a rich source of dietary fiber, proteins, and carbohydrates, which are essential macromolecules for proper nutrition and growth. Wheat is grown on around 218 million hectares of agricultural land, providing food for nearly 21% of the population worldwide [3]. In Pakistan, 80% of farmers cultivate wheat on 9.0 million hectares as their primary food and income source. To meet the needs of the 9.7 billion people predicted by 2050, it is imperative to intensify wheat productivity [4]. Nevertheless, biotic and abiotic factors are the major handicaps responsible for the decline in wheat productivity. So, the urgent need is to have a higher grain yield, and this can be achieved by employing modern nanotechnology approaches, such as the foliar implications of nanobiochar suspension (NBS), that may increase crop productivity and grain quality [5].

In Pakistan, wheat cereal grain yield is comparatively low compared to other wheat-growing countries around the globe. The foremost reason behind reduced crop yield is the high soil pH, calcareous nature, and low soil organic matter content [6]. To cope with these challenges, optimal nutrient supply is mandatory, either through soil application or foliar spray. However, the foliar supplementation of NBS predominantly increases wheat productivity and the satisfaction of the demand for wheat as a primary food source in various countries around the world [7]. Additionally, appropriate crop growth can be achieved through proper agronomic practices and the management of fertilizer implications, which ultimately improve crop productivity by regulating the metabolic activities of cells [8].

In Pakistan, the wheat variety Zincol was released in 2016 by the Pakistan Agriculture Research Council (PARC) in collaboration with the HarvestPlus project. The wheat variety “Zincol-2016” maintains a higher grains’ zinc concentration than the previously released wheat varieties [9]. Farmers have widely adopted and are still cultivating this variety on a large scale due to its higher grain yield enriched with zinc. Zincol-2016 holds significant potential to enhance consumers’ zinc intake, which is necessary to maintain zinc body requirements for most of the world’s population [10].

Poor soil fertility and less efficient use of synthetic fertilizers are the main reasons for reduced crop productivity [11]. Nutrient deficiency drastically harms wheat grain yield because it severely hampers or slows several physio-biochemical activities within the cells. It also disrupts cell homeostasis and hormonal regulation by inducing alterations in cellular respiration and various other metabolic processes [12]. Biochar is a rich source of carbonaceous material generated through several thermochemical events in lignocellulosic biomass [13]. Through the exploitation of a size-reducing approach up to the nano-meter level, biochar can be amended into nano-biochar (NB) form. Because of its larger surface area, surface specificity, greater catalytic activity, higher stabilization, improved porosity, structural uniqueness, and particular surface-active sites, NB has triggered promising avenues for modulating morpho-physiological and biochemical attributes in germinating crops. Nano-biochar reinforces efficient transportation, regularization, and absorption of essential macro- and micronutrients. Additionally, the implications of NB significantly enhance the physiological and biochemical activities of wheat through the mitigation of various abiotic stresses and the elimination of reactive oxygen species [14]. The foliar application of NBS is a more efficient and promising approach compared to soil application, where nutrients are required in greater quantity [15]. 

In the agricultural sector, nanoscience technology can lead to a significant increase in wheat productivity [16]. Nano-technological tools aid in acquiring a controlled release of nutrients to gain higher crop yields and increase fertilization efficiency [17]. Therefore, this research was carried out to find the most promising NBS foliar treatment for wheat at the end of tillering/beginning of leaf sheath elongation to regulate physio-biochemical activities that would ultimately improve wheat productivity.

## 2. Results

### 2.1. Growth Parameters

**Figure 1:**
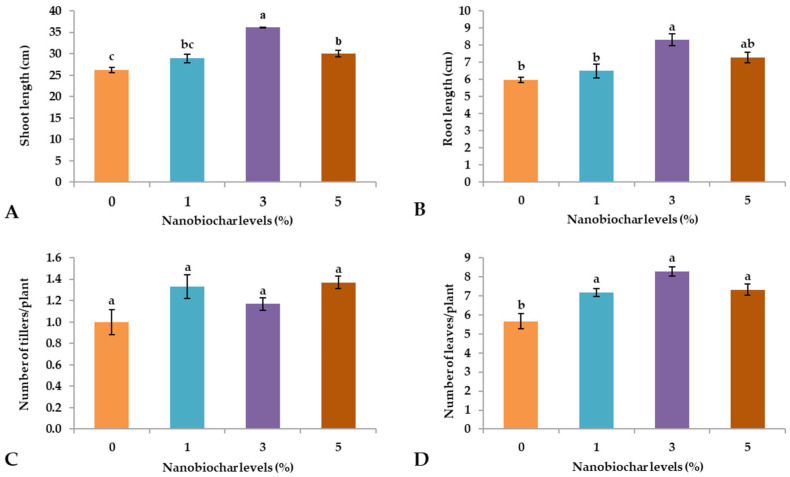
Effect of nano-biochar suspension (NBS) treatment on (**A**) shoot length, (**B**) root length, (**C**) number of tillers, and (**D**) number of leaves. Mean values of three replications for each treatment; different letters depicted significant variations among the applied treatments (*p* ≤ 0.05).

The results of the current investigation revealed that the foliar application of NBS significantly (*p* ≤ 0.05) affected the shoot length (SL) of developing wheat seedlings, with the average longest shoots (36 cm) recorded from the seedlings treated with 3% NBS, while lower values were observed with the application of 5, 1, and 0% NBS (30, 29, and 26 cm, respectively; Figure 1A, Table 1a).

The root length (RL) of the wheat seedlings (Figure 1B) resembled the trend observed for the length of the shoot, with 3% NBS resulting in the average longest roots (8.3 cm), and the shortest root length was observed in seedlings treated with 0% NBS (5.97 cm).

Regarding the number of tillers per plant (NOT), the application of NBS did not show any significant change regardless of treatment (Figure 1C). In contrast, the number of leaves per plant (NOL) was significantly (*p* ≤ 0.05) enhanced by foliar treatments with NBS, regardless of the percentage of NB, while 0% treatment showed the lowest number of leaves (5.67; Figure 1D). 

**Figure 2:**
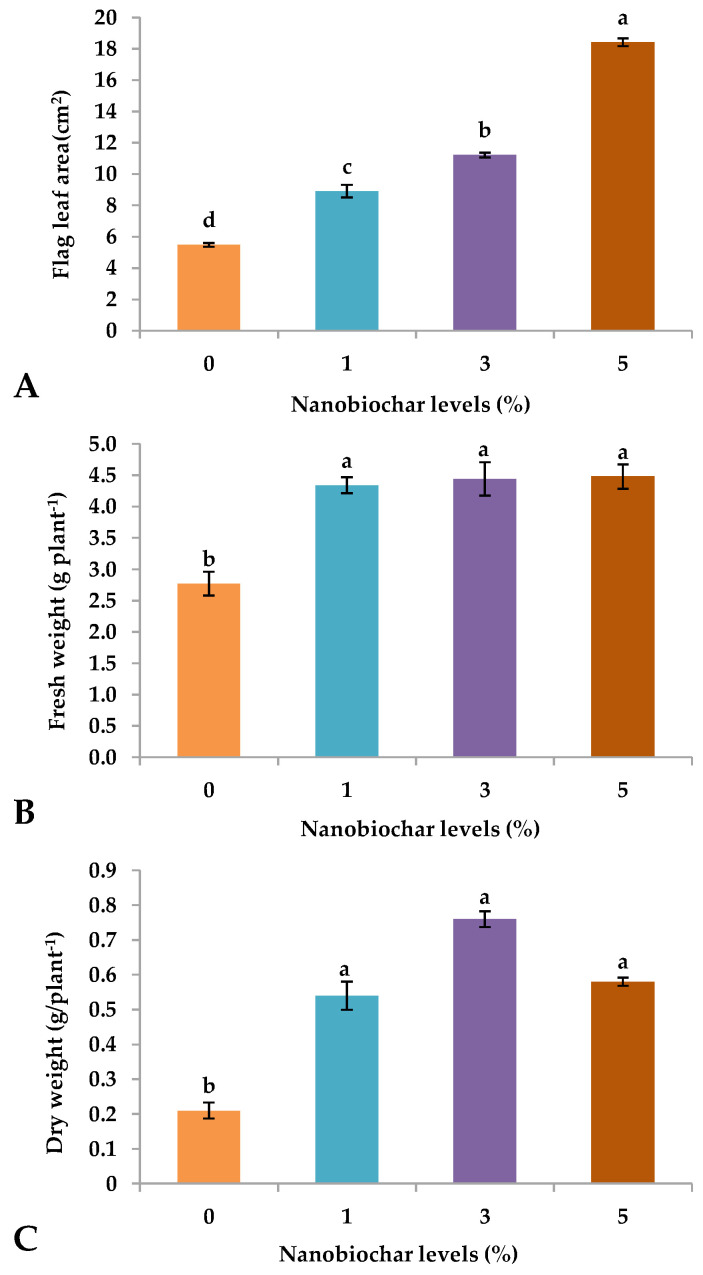
Effect of nano-biochar suspension (NBS) treatment on (**A**) leaf area, (**B**) total fresh weight/plant, and (**C**) total dry weight/plant. Mean values of three replications for each treatment; different letters depicted significant variations among the applied treatments (*p* ≤ 0.05).

The exogenous application of NBS on growing wheat at 5% significantly (*p* ≤ 0.05) increased the leaf area per plant (18.42 cm), and a declining trend was followed by 3 > 1 > and 0% NBS (Figure 2A). Furthermore, foliar sprays of NBS significantly (*p* ≤ 0.05) enhanced the fresh (FW) and dry weight (DW) of growing seedlings, regardless of the percentage of NB, while the 0% treatment exhibited the lowest values (Figure 2B,C).

### 2.2. Biochemical Alterations

**Figure 3:**
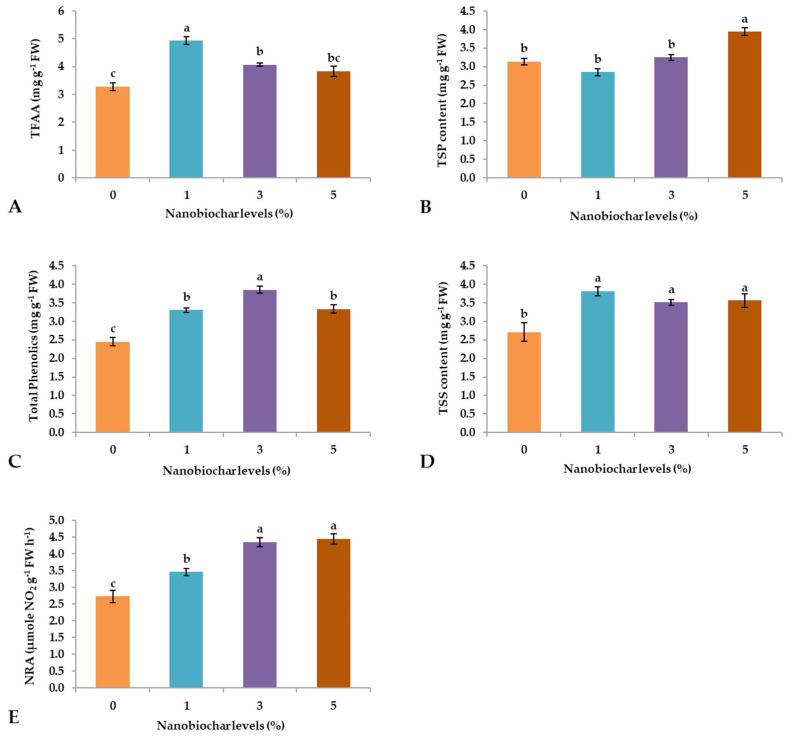
Effect of nano-biochar suspension (NBS) treatment on (**A**) TFAA contents, (**B**) total soluble proteins, (**C**) total phenolics, (**D**) total soluble sugars, and (**E**) NRA activity. Mean values of three replications for each treatment; different letters depicted significant variations among the applied treatments (*p* ≤ 0.05).

The contents of total free amino acids (TFAA) were significantly influenced (*p* ≤ 0.05) by NBS treatments, especially when the seedlings were treated with 1% NBS, and TFAA reached an average value of 4.93 mg g^−1^ FW (Figure 3A). The application of 3 and 5% NBS resulted in lower values than 1% NBS (4.07 and 3.83 mg g^−1^ FW, respectively), and 0% showed the lowest TFAA content (3.28 mg g^−1^ FW; Figure 3A). Regarding total soluble protein (TSP), the highest average value was observed for 5% NBS, while all other treatments were similar to each other (Figure 3B). Moreover, the TSP content was significantly higher in plants treated with 3% NBS (3.85 mg g^−1^ FW), followed by 1 and 5% suspensions, and the lowest content was recorded from the treatment with 0% (2.45 mg g^−1^ FW). Moreover, total soluble sugars (TSSs) content was significantly enhanced by foliar treatment with NBS, regardless of their percentage, while 0% treatment resulted in the lowest TSSs content (2.71 mg g^−1^ FW; Figure 3D). The highest significant levels of nitrate reductase activity (NRA) were observed in plants sprayed with 3 and 5% NBS, followed by 1% treatment, while the lowest activities (2.73 µmole NO_2_ g^−1^ FW h^−1^) were recorded in plants treated with 0% NBS (Figure 3E).

### 2.3. Physio-Biochemical Attributes

**Figure 4:**
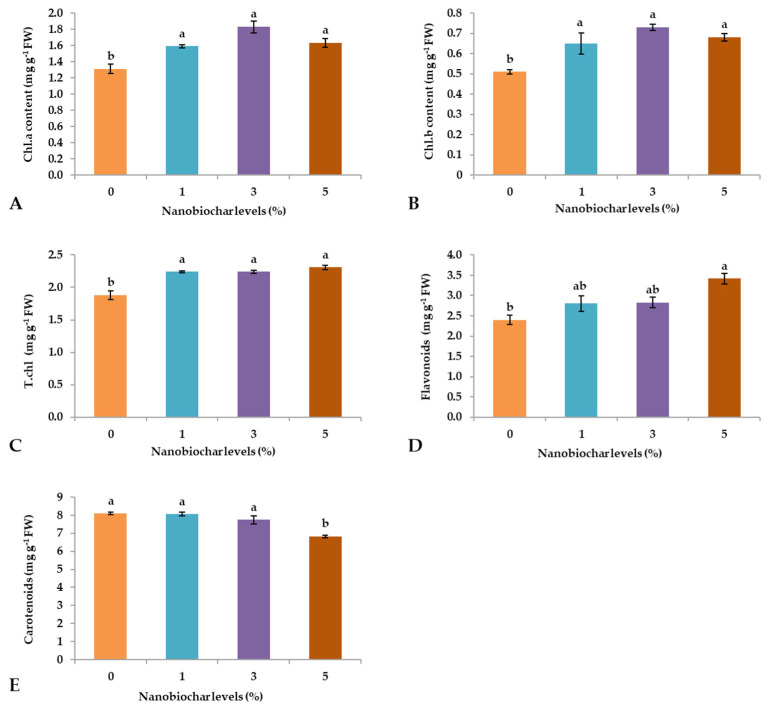
Effect of nano-biochar suspension (NBS) treatment on (**A**) chlorophyll a content, (**B**) chlorophyll b content, (**C**) total chlorophyll content, (**D**) flavonoids content, and (**E**) carotenoids content. Mean values of three replications for each treatment; different letters depicted significant variations among the applied treatments (*p* ≤ 0.05).

Among physiological attributes, chlorophyll a (Chl.a) content was significantly (*p* ≤ 0.05) affected by the foliar application of NBS. The highest Chla concentration was observed in plants sprayed with 3% NBS (1.83 mg g^−1^ FW), followed by treatments with 1 and 5% NBS showing similar contents (1.59 and 1.63 mg g^−1^ FW, not significantly different), while the lowest Chla content was recorded from plants treated with 0% (1.3 mg g^−1^ FW; Figure 4A). Regarding chlorophyll b (Chl.b), its content was significantly influenced (*p* ≤ 0.05) by the foliar treatment with NBS, regardless of the percentage, and the lowest values were recorded from plants grown under control conditions (0%; Figure 4B). The highest flavonoid content (Flav) was recorded from plants treated with 5% NBS (3.42 mg g^−1^ FW), followed by treatment with 3 and 1% NBS (2.83 and 2.8 mg g^−1^ FW, not significantly different), and treatment with 0% showed the lowest content (2.4 mg g^−1^ FW; Figure 4D). Finally, the carotenoid contents ranged from 6.82 to 8.09 mg g^−1^ FW but were not significantly affected by any treatment (Figure 4E). 

### 2.4. Nutrient Contents 

**Figure 5:**
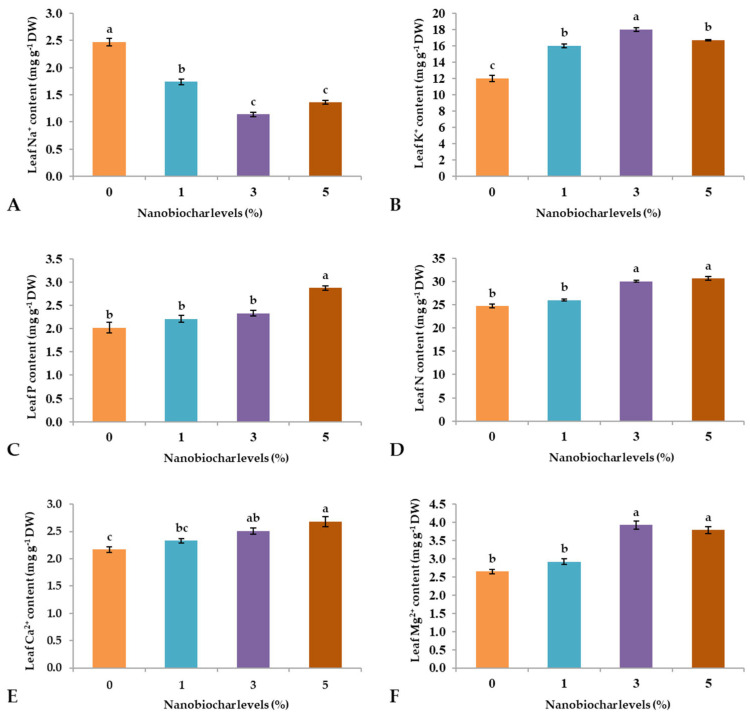
Effect of nano-biochar suspension (NBS) treatment on (**A**) sodium content, (**B**) potassium content, (**C**) phosphorus content, (**D**) nitrogen content, (**E**) calcium content, and (**F**) magnesium content. Mean values of three replications for each treatment; different letters depicted significant variations among the applied treatments (*p* ≤ 0.05).

The foliar treatments significantly affected the concentration of Na, K, P, N, Ca, and Mg in wheat leaves (Figure 5, Table 1b). The highest sodium concentration was retained by plants sprayed with 0% NBS (2.47 mg g^−1^ DW), while the lowest was recorded from plants treated with 3 and 5% NBS (Figure 5A). The highest K content was retained by leaves of plants sprayed with 3% NBS (18 mg g^−1^ DW), followed by treatments with 5, 1, and 0% NBS (Figure 5B). Phosphorous and Ca showed a similar trend, with 5% NBS resulting in the highest content, followed by treatments with 3, 1, and 0% NBS (Figure 5C,E). Finally, N and Mg showed quite similar trends, with 3 and 5% NBS resulting in the highest concentration, followed by 1 and 0% NBS (Figure 5D,F). 

### 2.5. Principal Component and Heatmap Analysis

**Figure 6:**
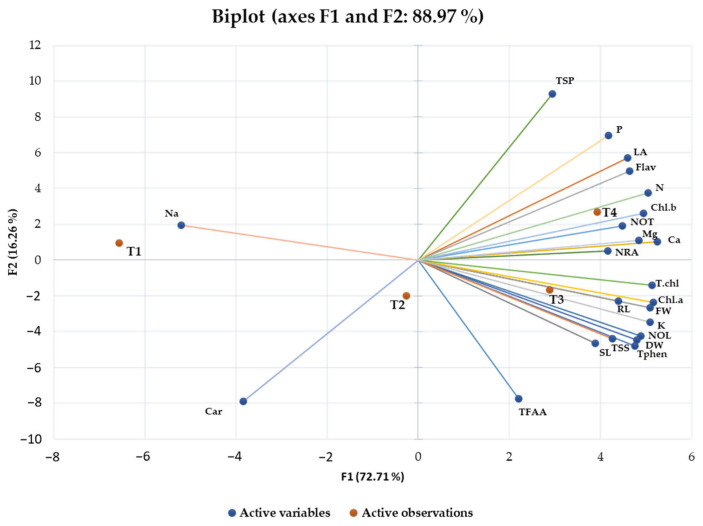
Biplot (score values) constructed through of all the studied morpho-physiological, biochemical, and nutrient contents in germinating wheat seedlings grown under four different treatments of nano-biochar suspension.

**Figure 7:**
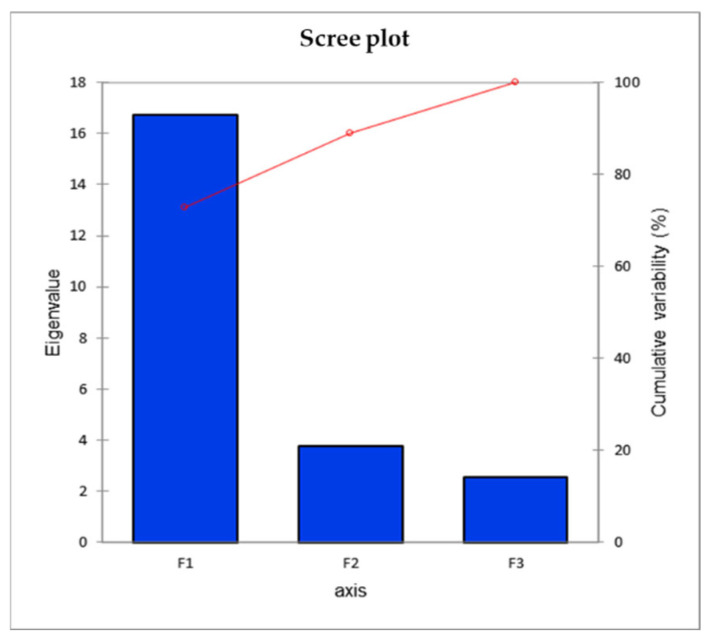
Blue bars (scree-plot) show eigenvalues of all the principal components (PCs) and red line show cumulative variability percentage.

Principal component analysis is used to evaluate the variations among all the parameters studied and assists in more easily understanding the larger and more complex data set. To determine the extent of variation in all morpho-physiological, biochemical, and nutrient contents studied under particular NBS treatments, a biplot was developed using PCA analysis using XL-STAT software (version 2018). The PCA biplot was constructed among the first two principal components (PC-1 and PC-2) since they revealed the maximum variation compared to PC-3 (Figure 6). Additionally, the eigenvalues bar graph (scree plot) showed that PC-1 and PC-2 contributed to the higher variation content. So, the biplot analysis was conducted among the first two principal components of PCs (Figure 7). In the constructed biplot, PC-1 and PC-2 contributed 72.71 and 16.26%, respectively, to the total variability. A longer vector from the centroid to the peripheral area means more variation toward the total variability. All parameters studied were positively correlated with T3 (3% NBS treatment) and T4 (5% NBS), while 0% (T1) was negatively correlated with the content of Na and carotenoids. Therefore, the PCA analysis confirmed that the most promising treatments were the application of 3 and 5% NBS (Figure 6). 

**Figure 8:**
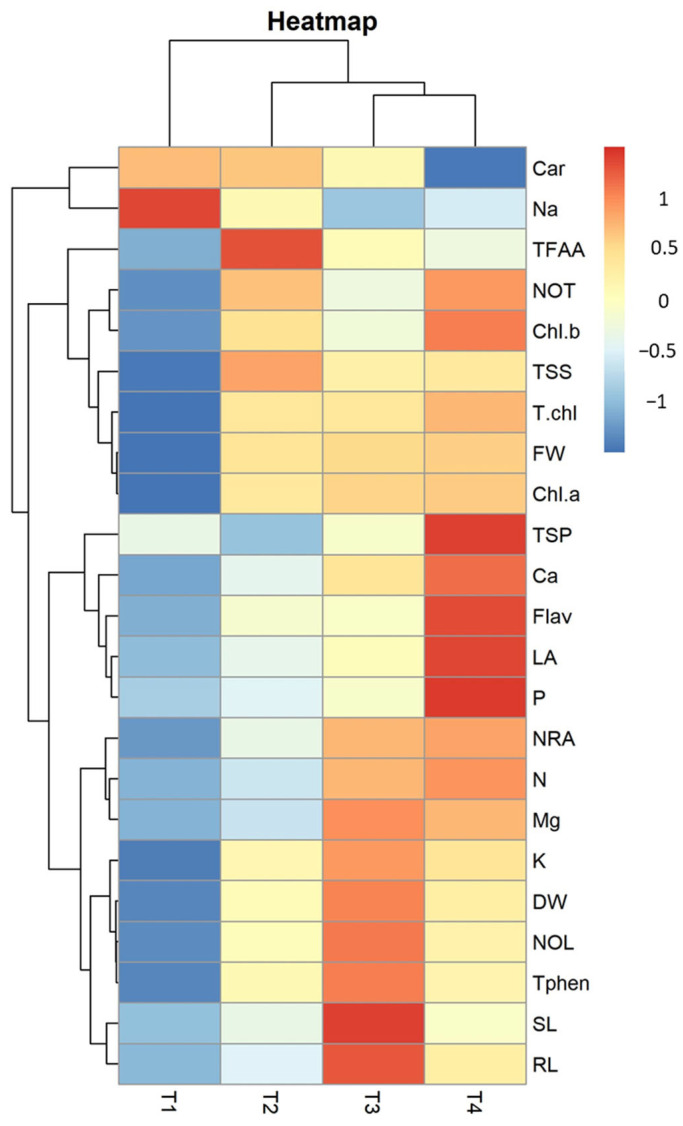
Heatmap correlation analysis for all the studied morpho-physiological, biochemical, and nutrient contents in growing wheat seedlings under different treatments of nano-biochar suspension.

Moreover, heatmap analysis was performed using RStudio software (version 4.0.3) to assess the promising effectiveness between foliar-sprayed NBS and the studied morpho-physiological, biochemical parameters, and nutrient contents (Figure 8). Heatmap analysis demonstrated three main clusters in which all traits were distributed, along with four different treatments. More favorable associations were observed between Treatment-3 (3% NBS) and several important traits, such as shoot length (Figure 1A), root length (Figure 1B), total phenolic content (Figure 3C), total dry biomass of plants (Figure 2C), NRA activity (Figure 3E), potassium (Figure 5B), nitrogen (Figure 5D), and magnesium contents (Figure 5F). Treatment-4 (NBS 5%) was effective in improving some traits, such as flavonoids (Figure 4D), leaf area (Figure 2A), and calcium content (Figure 5E), while its negative association was recorded with carotenoid content (Figure 4E). However, Treatment-1 (NBS 0%) and Treatment-2 (NBS 1%) showed a negative correlation in favor of all of the attributes mentioned above.

## 3. Discussion

To achieve higher crop productivity, considerable improvements in morpho-physiology, biochemical activities, and crop nutrient uptake are mandatory, as these changes play a crucial role in combating oxidative stress by enhancing antioxidant production. Compared to bulk biochar, nano-sized biochar is a distinct product due to its surface characteristics (larger surface area), elemental composition, and carbon stability. This heterogeneous material at the nanoscale level is effective in improving growth and crop yield. In the present research, we exogenously applied NBS in different concentrations to determine the optimal NBS rate that enhances plant growth and development and to explore physio-biochemical activities and nutrient uptake alterations.

The exogenous application of NBS significantly improved the growth of wheat plants during the early vegetative stage [18]. A significant increase in shoot length (Figure 1A), root length (Figure 1B), number of leaves per plant (Figure 1D), flag leaf area (Figure 2A), fresh weight per plant (Figure 2B), dry weight per plant (Figure 2C) evidenced the effectiveness of NBS. In general, an improvement in growth characteristics was recorded in plants that were foliarly sprayed with 3% NBS. Another study revealed that exogenous application of NBS effectively improved plant growth and development by boosting plants’ metabolism, respiratory activity, antioxidant activity, and photosynthetic efficiency [19]. Nevertheless, foliar supplementation with NBS or nano-nutrient solution (NNS), enhances plant growth by improving root length, shoot length, number of leaves, number of tillers, and leaf surface area by activating key enzymes, genes, and proteins. Previous research conducted by Shani et al. [15] found that foliar spray nano-biochar suspension enhances the fruit weight and number of fruits per tree. 

The foliar application of NBS demonstrated growth improvements compared to the soil application of NB [20]. Earlier findings highlighted the positive impact of NB on plant growth, emphasizing its superiority over inorganic fertilizers due to its gradual nutrient release, fulfilling the requirements for normal growth [21]. Foliar-sprayed NBS enters the leaves via stomatal openings, moves through apoplastic and symplastic pathways, penetrates leaf cells, and can be transported to various plant organs [22]. Therefore, NBS contributed to the increase of the levels of N, P, K, Mg, and Ca in the leaves and, at the same time, reduced the Na content (Figure 5). With regards to the latter aspect, the foliar application of NBS could have had effects at the root level by up-regulating Na^+^/H^+^ antiporters, thus helping in the compartmentation of Na^+^ in the vacuoles of the root cells or outside the roots [23]. Additionally, overaccumulation of sodium ions within cells leads to the induction of salinity stress, which may cause injury to the cell membrane and shows an antagonistic relationship with K uptake in plant cells [24]. Therefore, maintaining sodium concentration at an optimal level is mandatory for the proper functioning of other important ions, such as K intake inside the cell [25]. Earlier findings by Sári et al. [5] stated that the foliar application of NBS played an indispensable role in maintaining the Na/K ratio for the optimal working of Na/K pumps and improved the NRA, which is a main source for the formation of NADPH, essential in regulating photosynthetic activities, the electron transport chain, and several other important metabolic activities. Moreover, under harsh environmental conditions, the foliar supplementation of NBS raises the N, P, and K concentrations within the plant cells, leading to enhanced metabolic activities and the synthesis of crucial amino acids and key proteins required to activate the genes responsible for stress tolerance in growing plants [26]. An increase in phosphorus and nitrogen contents leads to an improvement in the plant’s photosynthetic efficiency, which is essential for energy-generating processes in plants. Consequently, optimal root growth is observed, which enhances the nitrogen fixation ability of leguminous plants [27] and the appropriate functioning of photosystems [28]. While the accumulation of calcium and magnesium in plant cells under stress conditions neutralizes the content of organic acids and improves the ability to absorb other nutrients from the roots [29]. Even in the present study, we recorded an increase in NRA activity with the application of NBS, especially with a higher percentage of NBS (Figure 3E). The increase in NRA is required for the assimilation of nitrogen absorbed by plants, which regulates the modulation of the expression pattern of the nitrate reductase genes and the transcription factors necessary for catalyzing appropriate physiological processes [30]. 

The results related to photosynthetic pigments [31] are consistent with previous research activities [32], which reported that the foliar treatment of NBS plays a crucial role in improving the pigment content in *Daucus carota*. Similarly, another study conducted by Mubashir et al. [33] revealed that foliar application of a biochar-based nano-nutrient solution increased photosynthetic pigments and flavonoid contents by improving antioxidant enzymes and retarding oxidative stress damage in *Solanum lycopersicum* under drought stress conditions. Moreover, in the current study, phenolic content (Tphen) was positively affected by the application of NBS, especially at 3% concentration (Figure 3C). Despite this, Shafiq et al. [18] unveiled that the copper oxide nanoparticles foliar treatment plays a crucial role in declining the excessive production of reactive oxygen species by increasing the production of antioxidant enzymes under harmful environmental conditions.

The PCA indicated that treatments with 3 and 5% NBS showed the highest values of positive features of wheat seedlings such as shoot and root length (Figure 1A,B), fresh weight (Figure 2B), dry weight (Figure 2C), number of leaves (Figure 1D), total soluble sugars (Figure 3D), total phenols (Figure 3C), and chlorophyll contents (Figure 4). Moreover, heatmap analysis showed that 3% NBS promisingly increased several plant attributes, such as total soluble sugars, total phenolics, chlorophyll contents, and key nutrients, i.e., K, P, N, Ca, and Mg content (Figure 5). In contrast, 0% NBS treatment resulted in the accumulation of Na (Figure 5A), possibly due to the lack of NB in plant tissues that can mediate the adsorption and release of nutrients. In fact, the soil application of biochar has already been investigated, showing plant-promoting effects and increased moisture retention, mediation between the adsorption and the release of nutrients, and the inhibition of pathogens [34,35]. Therefore, the foliar supplementation of biochar, and especially NB, could result in the same positive effects when applied to the soil because this organic matter is intimately impregnated within the tissues and cells of plants.

## 4. Materials and Methods

### 4.1. Experimental Design and Soil Analyses 

The field experiment was conducted from October 2021 to April 2022 at the botanical research area of the University of Lahore, Pakistan, in a plot that had been fallow for the previous four years. The soil was sampled before wheat sowing to represent the soil conditions. Soil samples were collected at a depth of 0–30 cm using an auger and following a W scheme. The soil texture was obtained using the method described by Dewis and Freitas [36], while the main physico-chemical characteristics were obtained according to the methods by Jackson [37], as described by Shani et al. [38]. The soil analyses are reported in Table 2.

Fertilizers were applied as recommended for wheat by the Agriculture Department: before sowing, the entire field was fertilized with 50 kg of diammonium phosphate (18 N, 46 P_2_O_5_) ha^−1^. The Zincol-2016 wheat variety was then sown while maintaining a row spacing of 30 cm and a plant spacing of 15 cm. Finally, 100 kg urea (46 N) and 50 kg sulfate of potash (50 K_2_O, 18 S) ha^−1^ were uniformly distributed at three times between stages 10 and 11 of the Feekes’ scale for the cereal growth (roughly between the stage of heading and the beginning of ripening) [39]. 

The experiment site was divided into 12 plots distributed in a completely randomized block design, obtained from three replications for each treatment. The four treatments were as follows: 0% NBS, wheat plants were foliar sprayed with distilled water at stages 2 and 3 of the Feekes’ scale, corresponding to the end of tillering/beginning of leaf sheath elongation; 1%, 3%, or 5% NBS, wheat plants were foliar sprayed with nanobiochar suspensions in distilled water having the respective concentrations at the same crop stages mentioned above. We decided to use these concentrations because they are in the range of the usual application of foliar fertilizers.

During the study period, temperatures ranged from a maximum of 30 °C to a minimum of 4 °C. The total rainfall was 600 mm, and the wheat crop received four additional irrigations, each providing 75 mm of water.

Finally, biometric parameters were obtained by collecting five plants from each replication at stages 4 and 5 of the Feekes’ scale.

### 4.2. Characterization of Nanobiochar Suspension

The nanobiochar used in the present study was obtained from the College of Life Sciences, Northwest A&F University (Northern Campus), Yangling, Shaanxi, China. The descriptive study on the fabrication of NBS has already been published by Khaliq et al. [32] and Shani et al. [15]. The scanning electron micrography of NB depicted a variety of spherical, smooth, and flake-like morphologies. Furthermore, NB was characterized by several blackish spots that indicated enriched C areas, whereas the crystallized regions revealed an elevated content of Ca. The elemental composition of NB was the following: C 62.5%, O 28.8%, H 1.92%, N 0.19%, P 0.15%. Using ICP-AES spectrometry, several other elements were found in trace in NB including Ca, Na, Si, K, Fe, Al, Mg, Sr, Cr, and Ti. Finally, NBS showed pH 10.3 and EC 3.02 dS m^−1^. 

### 4.3. Estimation of Physio-Biochemical and Nutrient Contents at the Seedling Stages

Physio-biochemical alterations were assessed one month after the foliar application of water and NBS on wheat seedlings (stages 4 and 5 of the Feekes’ scale for cereal) [39]. Nitrate reductase activity (NRA) was determined using the protocol by Sym [40] and total free amino acids (TFAA) through the Hamilton and Van Slyke method [41]. Lowry et al. [42] protocol was used for the estimation of total soluble proteins (TSP), while total soluble sugars (TSSs) were determined using the Riazi et al. [43] procedure. The total flavonoids (TFL) content was calculated following the Pekal and Pyrzynska [44] method, and the pigments analyses (total chlorophylls and carotenoids, chlorophyll A and B) were carried out using the Arnon [45] and Davies [46] protocols. Dried leaf material was used to estimate the nutrient contents. For nutrient content analyses, flag leaves were collected one month after the NBS application. The harvested leaves were rinsed twice with tap water and once with distilled water. The leaves were dried in a forced air oven running at 70 ± 2 °C for 72 h. The dried powder of ground leaf after digestion [38] was used for nutrient analysis; that is, the leaf phosphorus content (P) was determined following Jackson’s method [37], total N was assessed using the micro-Kjeldhal method [47], and Na and Ca were determined using a Jenway-PFP7 flame photometer (Leicestershire, UK).

### 4.4. Statistical Analyses

All data were analyzed using analysis of variance (ANOVA) to determine the significance level at (*p* ≤ 0.05). The mean data of all the results were compared at a probability level of 5% by performing Tukey’s test using Statistix 8.1. Additionally, the principal component analysis and heatmap analysis were performed using R software (version 4.3.0).

## 5. Conclusions

The current investigation suggests that the foliar application of NBS is effective in enhancing the growth of wheat seedlings. Among the treatments studied, the 3% NBS showed the best results in terms of plant growth and physio-biochemical activities. The application of 5% NBS is also promising since it resulted in almost the same beneficial effects as those achieved by 3% NBS, although some parameters studied were below those recorded from the application of 3% NBS. Therefore, more studies are needed to recognize the right rate/dose of NBS and to check the number of applications of NBS throughout the crop cycle. 

## Figures and Tables

**Table 1:** (**a**). Means sum of squares for all the analyzed morpho-physiological and biochemical attributes under different foliar treatments of nano-biochar suspension (NBS). (**b**). Means sum of squares for nutrient contents analyzed under different foliar treatments of nano-biochar suspension (NBS).

(a)																		
Source	DF	SL	RL	FW	DW	NOL	NOT	LA	NRA	TFAA	TSP	T.Phen	TSSs	Flav	Chl.a	Chl.b	T.chl	Car
Treatment	3	53 ***	3.07 ***	2.05 **	0.15 *	3.49 *	0.08 ^ns^	89.94 ***	1.97 **	1.36 **	0.64 **	1.007 ***	0.68 **	0.52 ***	0.137 **	0.022 *	0.11 **	1.04 **
Error	6	4.10	0.03	0.12	0.013	0.25	0.02	0.14	0.06	0.06	0.03	0.001	0.01	0.002	0.004	0.003	0.004	0.06
Total	11																	
**(b)**																		
**Source**	**DF**	**Na**	**K**	**P**	**N**	**Ca**	**Mg**											
Treatment	3	1.02 ***	20.06 ***	0.39 **	25.52 ***	0.14 **	1.21 **											
Error	6	0.006	0.22	0.008	0.07	0.01	0.02											
Total	11																	

Showing significant differences among all the applied NBS foliar treatments *p* > 0.001 ***, *p* < 0.01 **, (0.01 < *p* > 0.05) *, *p* > 0.05, ns (non-significant); DF, degrees of freedom; SL, shoot length; RL, root length; FW, total fresh weight; DW, total dry weight; NOL, number of leaves; NOT, number of tillers; LA, leaves area; NRA, nitrate reductase activity; TFAA, total free amino acids; TSP, total soluble proteins; T.Phen, total phenolic contents; TSSs, total soluble sugars; Flav, flavonoids contents; Chl.a, chlorophyll a content; Chl.b, chlorophyll b content; T.chl, total chlorophyll; and Car, total carotenoids contents. Na, sodium contents; K, potassium content; P, phosphorus content; N, nitrogen content; Ca, calcium content; and Mg, magnesium content.

**Table 2:** Minimum and maximum values of physical and chemical characteristics of the soil used for the trial; nil, zero.

Soil Characteristics	Values
Physiological characters	
Soil texture	Clay loam
Chemical characters	
Saturation percentage (%)	40–41
ECe (dS m^−1^)	1.7–1.9
pH	7.5–7.8
Organic matter (%)	0.5–0.7
Ca+Mg (meq L^−1^)	2.6–4.8
CO_3_ (meq L^−1^)	Nil
HCO_3_ (meq L^−1^)	2.5–4.8
NO_3_-N (mg kg^−1^)	3.5–4.5
Total nitrogen (g kg^−1^)	0.4–0.5
Available K (mg kg^−1^)	75–80
Available P (mg kg^−1^)	1.5–2.8

## Data Availability

Data is contained within the article.
